# Comparative Bioavailability of DHA and EPA from Microalgal and Fish Oil in Adults

**DOI:** 10.3390/ijms26199343

**Published:** 2025-09-24

**Authors:** Eileen Bailey, Jérôme Wojcik, Maike Rahn, Franz Roos, Anneleen Spooren, Kyoko Koshibu

**Affiliations:** 1 Health, Nutrition and Care, dsm-firmenich, 4303 Kaiseraugst, Switzerland; maike.rahn@dsm-firmenich.com (M.R.); franz.roos@dsm-firmenich.com (F.R.); anneleen.spooren@dsm-firmenich.com (A.S.); 2 Data2time Sàrl, 1202 Geneva, Switzerland; jerome.wojcik@data2time.com; 3 Faculty of Business, Economics, and Informatics, University of Zurich, 8006 Zurich, Switzerland

**Keywords:** algal oil, bioavailability, docosahexaenoic acid (DHA), eicosapentaenoic acid (EPA), fish oil, omega-3

## Abstract

Microalgae offer a promising sustainable source of essential nutrients, docosahexaenoic acid (DHA), and eicosapentaenoic acid (EPA). DHA and EPA are mainly obtained through fish, which are limited in number due to global climate change. Microalgal oil, on the other hand, has emerged as a sustainable and limitless source of DHA and EPA but the bioavailability of these nutrients has not been directly compared to fish oil. Therefore, the objective of this study is to evaluate and demonstrate the comparable DHA and EPA plasma bioavailability of microalgal and fish oil. We analyzed the plasma phospholipid levels of 74 adult men and women after 6 and 14 weeks of consuming omega-3 supplements derived from either microalgal or fish oil in a randomized double-blind placebo-controlled parallel-group clinical trial. We found that the bioavailability of DHA and EPA in plasma phospholipids from microalgal oil supplements are statistically non-inferior compared to fish oil supplements, despite the differences in production process and composition, indicating that microalgal oil is a reliable and bioavailable source of DHA and EPA.

## 1. Introduction

Docosahexaenoic (DHA, 22:6n-3) and eicosapentaenoic (EPA, 20:5n-3) acids are omega-3 long-chain polyunsaturated fatty acids (LC-PUFA), widely recognized as essential components of a healthy diet [1,2,3]. Beneficial effects of DHA and EPA are seen throughout life, from infancy to adulthood, for a wide range of health benefits, including cardiovascular, brain, eye, and immune health [1,4,5,6,7,8,9,10,11,12,13,14], some of which are the basis for authorized health claims by various regulatory authorities [3,15,16]. Despite the substantial body of evidence that adequate DHA and EPA intake is critical for health, much of the world population consumes lower amounts than recommended by the regulatory authorities and scientific experts, based on surveys conducted in 30+ countries [2,3,17,18]. Because the biosynthesis of DHA and EPA in humans is inefficient, these omega-3 fatty acids must be obtained through diet and supplementation [1,2,3,17,18]. Considering that experts and authoritative scientific organizations recommend intakes of 250–500 mg of DHA and EPA per day in adults [3,19,20,21], an annual human intake of well over 1 million metric tons is needed to meet global nutritional needs. This cannot be met with the current supply of fish-derived omega-3 oil [1,22,23,24,25,26]. Therefore, the need for an alternative source of DHA and EPA is imminent.

Walnut, flax, chia, canola, hemp, echium, and perilla seed oils are plant-based sources of the omega-3 fatty acids, such as alpha-linolenic acid (ALA); however, they offer minimal support for increasing blood DHA and EPA levels [27]. In contrast, microalgal oil has emerged as a sustainable source of omega-3 fatty acids [28,29,30,31]. Fish receive the majority of their DHA and EPA from their dietary intake of algae [1,32]. Large-scale fermentation of microalgae can yield ten times more LC-PUFA compared to fish from the same amount of biomass [33,34]. Furthermore, the aquatic environments for microalgae cultivation can offer more stable and consistent conditions than terrestrial ecosystems. As a result, seasonal and climate fluctuations have less impact on the growth of microalgae used to produce omega-3 fatty acids, ensuring the reliable production of these fatty acids [35,36]. Furthermore, microalgae cultivation can contribute to carbon balance, because some heterotrophic microalgae species use sustainable and renewable carbon sources as a substrate [37]. Consequently, microalgae biomanufacturing may offer a more reliable and sustainable source of the omega-3 fatty acids than other sources [35,38,39].

In the past, a direct comparison of the bioavailability of DHA and EPA in plasma phospholipids from microalgal vs. fish oil has not been conducted due to the lack of a commercially available microalgal oil with appreciable levels of EPA. The importance of evaluating the plasma levels of DHA and EPA separately lies in the fact that there are increasing number of reports suggesting subtle differences in the health benefits arising from DHA and EPA. For instance, during the first 1000 days of development, DHA in particular plays a pivotal role in supporting healthy brain and eye development. Longer-term benefits have been reported for cognitive performance and visual acuity [11,12,13]. For EPA, on the other hand, emerging scientific evidence suggests that it supports mood and well-being in adulthood [8,10,14].

Therefore, we aimed to gain a deeper understanding of the bioavailability of DHA and EPA from microalgal and fish omega-3 fatty acid supplements using the data obtained from our previously published randomized double-blind placebo-controlled clinical trial [40]. The current analysis is a re-evaluation of the plasma fatty acid data published by Maki et al. [40] to directly compare the effects of microalgal and fish oil supplementation on the plasma phospholipid EPA and DHA levels in these subjects. The microalgal oil, DHA-O, used in this study was derived from *Schizochytrium* sp., which unlike other microalgal oils, contains both DHA and EPA at a ratio of approximately 1:3 [38,39,40]. This deep-dive into the plasma phospholipid levels of DHA and EPA is critical because of the uncertainties regarding the fundamental differences in DHA and EPA bioavailability from different sources, composition matrixes, or chemical binding forms [35,41,42]. In fact, fish oils that are processed differently have been reported to have different DHA and EPA bioavailability [43]. Similarly, the bioavailability of different chemical binding forms of omega-3, ethyl ester, triglyceride, phospholipid, and free fatty acids have been reported to influence omega-3 bioavailability in rodents and humans [35,42,44,45,46,47]. Although both microalgal oil and fish oil contain omega-3 fatty acids in natural triglyceride forms [35], there is a fundamental need to evaluate the bioavailability of DHA and EPA derived from algal oil compared to fish oil, because the microalgal oil production, and thus composition, is innately different from fish oil [35,41].

Therefore, in this study, we tested our hypothesis that the microalgal omega-3 supplement is non-inferior in raising DHA and EPA plasma levels compared to the fish omega-3 supplement, regardless of the composition, when normalized to the supplement intake.

## 2. Results

### 2.1. Subjects

The dispositions of the subjects throughout the study are summarized as a CONSORT diagram in Supplementary Figure S1 and previously described in Maki et al. [40]. In brief, of the 93 subjects enrolled, seven subjects (2, 3, and 2 subjects from placebo, microalgal, and fish oil groups, respectively) discontinued prior to study completion due to adverse events, withdrawing consent, or loss-to-follow-up and were removed from analyses. The adverse events were mainly gastrointestinal in nature. Twelve additional subjects (3, 6, and 3 subjects from placebo, microalgal, and fish oil groups, respectively) were removed from the analyses due to non-compliant study-product consumption, weight loss, or consumption of fish oil within 2 weeks prior to screening. Thus, data from 74 subjects were included in this current analysis. The frequencies of any treatment-emergent adverse events were not significantly different among treatment groups (*p* = 0.082). There were no serious adverse events reported in the study.

Subjects were primarily non-Hispanic white males and females and had fasting triglyceride levels between 149 and 450 mg/dL. The demographics of the study subjects were evenly distributed among the placebo, microalgal oil, and fish oil groups in terms of mean age (52.5 ± 2.0, 52.6 ± 1.7 and 54.5 ± 2.0 years, respectively; *p* = 0.781), gender (50.0%, 51.4% and 60% female, respectively; *p* = 0.801), ethnicity (77.8%, 83.8% and 80.0% non-Hispanic white, respectively; *p* = 0.802), and body mass index (31.2 ± 0.7, 32.7 ± 1.0 and 31.9 ± 1.6 kg/m^2^, respectively; *p* = 0.544), as described previously [40].

### 2.2. DHA and EPA Intake from Food

**Figure 1:**
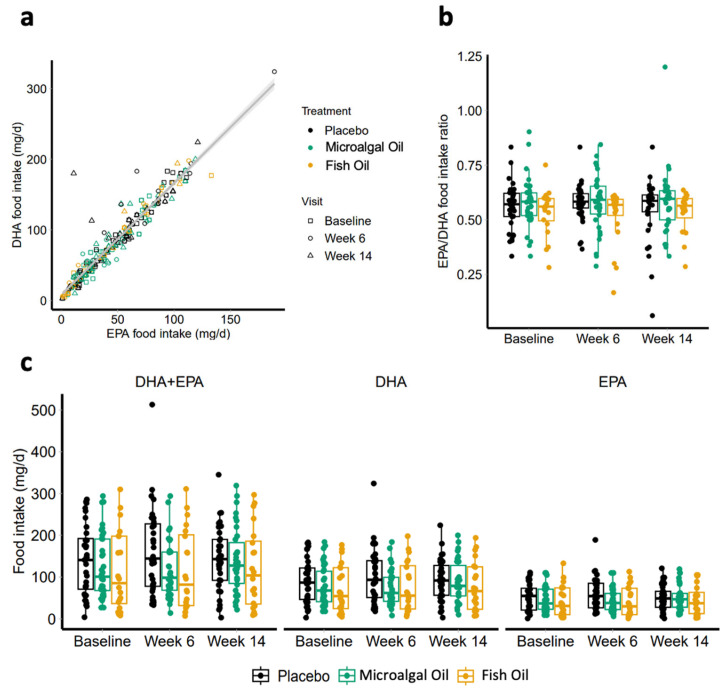
DHA and EPA intake from food. DHA and EPA intake from food was evaluated from food frequency questionnaires (FFQ) at baseline, week 6, and week 14. (**a**) There was a significant correlation between DHA and EPA food intake (r = 0.96). (**b**) The EPA to DHA ratio from food source was approximately 1:2, taking into account both weeks 6 and 14 data. (**c**) No significant differences in DHA and/or EPA from food intake were detected across groups. Box plots represent the median and 25th and 75th percentile. Each dot represents individual subjects. Black circles and lines = placebo group; green circles and lines = microalgal oil group; orange circles and lines = fish oil group.

DHA and EPA intake from food was assessed. We found that the total omega-3 fatty acid intake (EPA+DHA) was 133.1 ± 5.2 mg per day on average across the groups, and that DHA and EPA positively correlated with each other (r = 0.96) (Figure 1a). All groups had similar food intake of DHA and EPA at all time points based on the food frequency questionnaire (FFQ) (DHA+EPA *p* = 0.2248; DHA *p* = 0.2514; EPA *p* = 0.1719) (Figure 1c; Supplementary Table S2). The average ratio of EPA to DHA dietary intake was approximately 1:2 for all groups (Figure 1b; Supplementary Table S2). This ratio resembles the low EPA to DHA ratio of the microalgal oil used in this study. Interestingly, estimated DHA and EPA intake based on FFQ correlated with the baseline plasma levels of DHA and EPA (Supplementary Figure S2a–c; Pearson’s coefficient of correlation for DHA+EPA: R = 0.39, *p* = 0.0006; DHA: R = 0.35, *p* = 0.0023; EPA: R = 0.34, *p* = 0.0033).

### 2.3. Comparison of Plasma Phospholipid Levels

To compare the plasma phospholipid bioavailability of microalgal oil and fish oil, plasma DHA and EPA levels were normalized to the DHA and EPA supplement intake. The total plasma level of DHA and EPA increased significantly at week 6 and continued to be elevated at week 14 for both the microalgal (212 ± 106% and 213 ± 102%, respectively) and fish oil (161 ± 89% and 154 ± 61%, respectively) groups compared to the placebo group (10 ± 24% and 7 ± 31%, respectively) (*p* < 0.0001) (Supplementary Figure S3; Supplementary Tables S3–S5). The comparison of algal oil vs. placebo and fish oil vs. placebo at week 14 yielded power > 99.9%. As expected, the plasma phospholipid DHA and EPA levels did not change over time in the placebo group (*p* = 0.7316 and *p* = 0.8422, respectively). The plasma EPA/DHA ratios were 0.32 ± 0.01 (~1:3) and 0.59 ± 0.03 (~1:2) for the microalgal and fish oil groups, respectively, as compared to 0.28 ± 0.02 (~1:4) for the placebo group, taking into account both week 6 and 14 plasma levels. The higher plasma EPA to DHA ratio in the fish oil group reflected the higher EPA load of fish oil supplement compared to the microalgal oil supplement used in this study.

**Figure 2:**
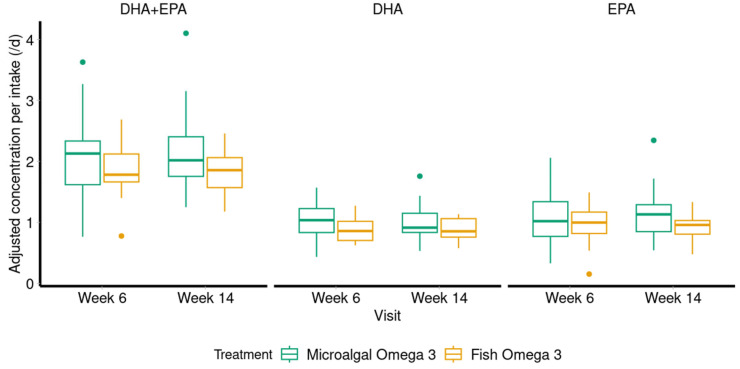
Comparison of plasma DHA and EPA bioavailability between microalgal and fish oils. The plasma bioavailability of total DHA and EPA (**left**), DHA (**middle**), and EPA (**right**) at week 6 and week 14 was compared between microalgal (green box plots) and fish (orange box plots) oil groups. The plasma levels were normalized to the dietary supplement of and EPA. Geometric mean ratio (GMR) and 90% confidence interval (CI) were for DHA+EPA = 112% [94–132%], DHA = 112% [96–130%], and EPA = 113% [90–142%], indicating that microalgal oil is comparable to fish oil. Box plots represent median and 25th and 75th percentile. The single green or orange dots represent outliers that are more than 1.5 times above or below the interquartile range.

To assess the non-inferiority of the plasma phospholipid bioavailability of DHA and EPA in microalgal vs. fish oil groups, the geometric mean ratio (GMR) was evaluated. We found that the total plasma phospholipid DHA and EPA bioavailability was non-inferior in microalgal vs. fish oil groups (GMR = 111% with 94–132% Confidence Interval (CI)) (Figure 2; Supplementary Table S6). Likewise, plasma DHA and EPA bioavailability considered separately were non-inferior between the groups (DHA GMR = 112% with 96–130% CI; EPA GMR = 113% with 90–142% CI). The one-sided power analysis with a 20% margin at α = 0.05 resulted in DHA+EPA a posteriori estimated power = 93%, DHA power = 96%, and EPA power = 80%. A complete plasma phospholipid composition as a percentage of total fatty acid is provided as a reference in Supplementary Table S7.

## 3. Discussion

The current study demonstrates that DHA and EPA plasma bioavailability of microalgal omega-3 oil and fish omega-3 oils is comparable after 6 and 14 weeks of consumption when normalized to the supplement intake. To our knowledge, the current study is the only study that has directly compared microalgal and fish oil and found similar DHA and EPA bioavailability considering the intake. The finding here is rather unique when considering that the fatty acid profiles of fish and microalgal oil are different (Supplementary Table S1), supporting the robustness of our finding, which is independent of the product source or composition. This current finding augments our previous report that demonstrated improvements in triglycerides and cholesterol levels in healthy adult participants after microalgal oil or fish oil supplementation [40].

The present study has several scientific strengths, including its randomized, double-blind, placebo-controlled design; the use of commercially available supplements to ensure real-world relevance; and the normalization of plasma DHA and EPA levels to intake, which appropriately addressed the different fatty acid compositions of the oils. The choice of plasma phospholipid levels as biomarkers of bioavailability is well supported, and the study’s 6- and 14-week duration was sufficient to reach steady state. Statistical analyses, including geometric mean ratios and non-inferiority testing, were robust, and the findings represent the first direct comparison of both DHA and EPA bioavailability from algal and fish oils. However, some limitations should be noted. The relatively small and unbalanced sample size, particularly in the fish oil group and the study population—primarily non-Hispanic white, overweight, middle-aged adults—limits generalizability. The reliance on self-reported dietary intake and supplement compliance introduces potential bias, and differences in EPA/DHA ratios between algal and fish oils complicate strict equivalence claims, despite normalization. Furthermore, only plasma phospholipids were assessed, without red blood cell or tissue measures that could provide additional insights. Some of these points are discussed in more detail in the following paragraphs.

In considering the current findings, it is important to note that the dose and ratio of EPA to DHA for microalgal oil and fish oil supplements are different. The fish oil supplement used in this study has a higher EPA to DHA ratio (3:2) compared to the microalgal oil (1:3). This difference in EPA to DHA ratio is consequently reflected in the higher absolute plasma phospholipid EPA concentrations in the fish oil group compared to the microalgal oil group, and the higher absolute plasma DHA concentration in the microalgal oil group compared to the fish oil group. The use of commercial products helped us to simulate a real-life scenario. This is the reason why the bioavailability comparisons were normalized to the supplement intake. Therefore, despite the differences in the absolute plasma DHA and EPA levels between the groups, we were able to demonstrate the non-inferiority of omega-3 triglycerides derived from microalgal vs. fish oil when supplement doses were taken into consideration. It is worth noting that there are two previous studies that have evaluated the nutritional equivalence of microalgal vs. fish source of omega-3 oils. Otto et al. have reported similar DHA bioavailability between tuna fish oil and microalgal oil in healthy non-pregnant females after 4 weeks of supplementation [48]. Similarly, Arterburn et al. demonstrated that DHA bioavailability was equivalent between microalgal oil and cooked salmon in healthy male and female adult volunteers after 2 weeks of supplementation or consumption [49]. Both studies could only evaluate DHA bioavailability because microalgal oil with a high EPA content was not commercially available at the time these studies were conducted.

Some studies report that EPA may be converted to DHA [50,51,52,53,54] and DHA supplementation is thought to slow down the metabolism and/or accumulation of plasma EPA, affecting the plasma concentrations [53,54]. Nevertheless, previous publications suggest that conversion is minor compared to the increase in DHA and EPA levels induced by dietary supplements [55,56]. In fact, the association between the DHA and EPA dose and plasma levels in our investigation would have been lost if the conversion had been sizeable. Therefore, the fact that the plasma phospholipid levels and doses were correlated in this study supports the previous findings that the DHA and EPA interconversions are minor events and that they do not seem to have a sizeable impact on plasma DHA and EPA bioavailability.

To compare DHA and EPA bioavailability, we evaluated the plasma phospholipid fatty acids as an exploratory objective of the clinical trial. The choice of plasma phospholipid DHA and EPA levels as a biomarker of bioavailability for studies lasting a few months is a standard practice, justified by previous publications using omega-3 supplements [57,58,59,60]. Plasma phospholipids are considered an acceptable surrogate marker for incorporation into red blood cells and tissue and have been used as a standard biomarker for DHA and EPA bioavailability in many studies in the past [57,58,59,60,61]. These studies have also evaluated plasma EPA and plasma DHA levels separately as a measure of bioavailability of omega-3 supplements. In our study design, it was reasonable to use plasma phospholipid levels of DHA and EPA as biomarkers of bioavailability because they have been reported to reach a steady state within 1 month and 2 months, respectively, and at steady state, plasma DHA and EPA levels correlated strongly with those in red blood cells (RBCs) [44,61]. Because our timepoints were 6 weeks and 14 weeks, it is appropriate to analyze plasma phospholipids. Furthermore, a previous report showed that between-subject variability in omega-3 levels were comparable when measured in RBCs and plasma following an acute single dose of omega-3 consumed with a standard breakfast [62]. This suggests that the variability observed in DHA and EPA plasma levels in our study (Supplementary Figure S3) is within the expected range and would likely have been similar had we assessed RBCs instead of plasma. The fact that we were able to significantly demonstrate that the consumption of fish and microalgal oils led to similar plasma phospholipid DHA and EPA levels supports the validity of using plasma phospholipid DHA and EPA as a biomarker of bioavailability.

Lastly, in the current study, we have compared the fish and microalgal sources of natural triglycerides. It is known that microalgal oil and fish oil can both produce natural triglyceride form of omega-3 [44]. Thus, the comparable plasma DHA and EPA bioavailability from such similar sources may seem unsurprising. The bioavailability, however, can be influenced by a number of factors. For example, a recent review on this topic by Martin Olmedo et al. suggests that the DHA and EPA bioavailability of fish oil can differ depending on the production process [43]. In addition, biochemical structure, matrix, and concomitant food intake can also influence the findings [63,64,65,66]. In rodents, DHA in free fatty acid, monoglyceride, triglyceride, and ethyl ester forms has been reported to have different bioavailability, which may depend on the concomitant intake of fatty food [45,46,47]. In contrast, fish-derived natural triglyceride, fish-derived ethyl ester, and krill-derived phospholipid showed the same DHA bioavailability in a human clinical trial [67]. Therefore, it cannot be taken for granted that just because microalgal oil and fish oil provides a source of omega-3, they would result in a similar bioavailability.

It is worth noting that our insights here are based on the observations during the course of supplement intake and cannot be directly extended to the long-term levels of DHA and EPA after the discontinuation of the supplementation. The half-life of DHA and EPA in plasma are reported to be between approximately 1.5 to 3 days after 1 day or 7 days of ingestion [68,69]. Interestingly, however, the theoretical half-life of DHA in the human brain is approximately 2.5 years, assuming that the DHA consumption of the brain is equivalent to the incorporation rate of 3.8 ± 1.7 mg/day [70]. Therefore, it would be interesting to evaluate the long-term changes in plasma and tissue levels of DHA and EPA after the discontinuation of the supplementation to better understand the long-term health benefits of DHA and EPA supplementation in future studies.

There are numerous studies reporting the plasma phospholipid DHA and EPA bioavailability of fish omega-3 oils [41,60,64], but only a few studies have reported the DHA bioavailability of microalgal oil [48,49] and none have done so for EPA. Therefore, we believe that this current analysis augments our previous findings that not only is microalgal oil able to provide cardiovascular health benefits by reducing the risk biomarkers [40] but also that this beneficial effect is most likely induced through the similarity in the DHA and EPA bioavailability of the microalgal omega-3 oil and the fish omega-3 oil.

## 4. Materials and Methods

### 4.1. Study Design

This study is a randomized double-blind placebo-controlled parallel-group clinical trial in healthy adult males and females and is registered in ClinicalTrial.gov (NCT01737099, 27 November 2012). The study was conducted at Biofortis Clinical Research (Addison, IL, USA) and Evanston Premier Healthcare Research (Evanston, IL, USA), according to Good Clinical Practice Guidelines, the Declaration of Helsinki (2000), and the United States 21 Code of Federal Regulations. The clinical protocol was approved by Quorum Review IRB (Seattle, WA, USA) (27507/1, 11 September 2012). The statistical analyses of the EPA and DHA bioavailability comparison described in this manuscript was conducted at Data2time Sàrl (Geneva, Switzerland).

The detailed protocol and primary and secondary endpoint descriptions to evaluate the biomarkers of cardiovascular risk have been published in our previous publication [40]. The randomization schedule was computer generated. Participants and all researchers involved in participant recruitment, data collection and entry, sample collection, processing, and analysis were blinded to the randomization groups until data entry was completed and the database was locked. This study focuses on the exploratory evaluation of the plasma DHA and EPA bioavailability comparison between algal and fish oils. Informed consent was obtained from all subjects and/or their legal guardian(s) prior to enrollment. In brief, subjects were randomized into three groups and stratified by statin use. The subjects consumed 4 × 1 g soft gel capsules per day with food, at the same time of the day, for 14 weeks. Each capsule contained the same amount of omega-3 or placebo (corn and soy oil). Subjects were instructed to maintain their habitual diet, sleep, and exercise patterns. Fasted blood samples were collected at baseline and weeks 6 and 14 (±2 days) to assess plasma DHA and EPA concentrations. Subjects were asked to take the clinical trial materials and fast for minimally 10 h before the blood draws. A self-reported FFQ was used to assess approximate intake of DHA and EPA from food sources at screening and at the two follow-up visits. The FFQ used in this study has been reported to significantly correlate with plasma phospholipid levels in multiple clinical studies [71,72,73]. Therefore, we were able to reliably estimate the daily DHA and EPA intake using the FFQ.

### 4.2. Study Population

The study population is described in the CONSORT diagram (Supplementary Figure S1) and in our previous publication [40]. In brief, a total of 93 healthy adults of ages 21 to 82 were enrolled: 36 in the placebo arm, 37 in the microalgal oil arm, and 20 in the fish oil arm. The number of subjects enrolled per group was decided based on the power calculation to achieve 80% power to detect a difference of 16.1% in the primary endpoint between control and algal oil groups, assuming a standard deviation of 24% (two-tailed α = 0.05). Subjects with a DHA intake of <200 mg/day (equivalent of <1 serving of fish/week) were included based on the validated FFQ [71]. This inclusion criterium is the standard threshold used in other omega-3 clinical trials. Participants were encouraged to refrain from consuming omega-3 FA supplements and omega-3 fortified foods throughout the trial and within 4 weeks prior to screening.

### 4.3. Supplements

The microalgal oil used in this study was derived from *Schizochytrium* sp. Both microalgal and fish oils were natural triglycerides. Subjects were asked to consume four soft gel capsules per day for 14 weeks. Each capsule of microalgal oil contained 164 mg EPA and 443 mg DHA (1:3 EPA to DHA ratio, DSM Nutritional Products, Columbia, MD, USA), and each capsule of fish oil contained 289 mg EPA and 205 mg DHA (3:2 EPA to DHA ratio, EPAX™ 6000 AS, Oslo, Norway). The placebo capsules contained a 1:1 ratio of corn oil and soy oil. The complete fatty acid composition of the fish and microalgal oils are described in Supplementary Table S1. The total of 656 mg EPA and 1772 mg DHA was consumed per day for microalgal oil group. The total of 1156 mg EPA and 820 mg DHA was consumed per day for fish oil group. These doses were the closest doses possible given the use of commercially available microalgal and fish omega-3 supplements. We believe that such differences are justifiable because the bioavailability of DHA does not plateau until approximately 2000 mg per day and that of EPA does not seem to plateau up to 5000 mg per day [55]. Capsule counts were self-reported at the end of each day to assess the overall mean compliance with supplement intake, defined as the ratio of consumed over expected dose. Subjects who were >80% compliant were included in the analyses.

### 4.4. Fatty Acid Analysis

Plasma phospholipid fatty acids were evaluated to determine DHA and EPA bioavailability, correcting for the differences in doses at baseline, week 6, and week 14. Plasma phospholipids are considered as an acceptable biomarker of DHA and EPA bioavailability and a good surrogate marker for incorporation into red blood cells and tissue, particularly for interventions lasting for 3 months [44,58,59,61]. Blood was collected in ethylenediaminetetraacetic acid (EDTA)-containing tubes and separated by centrifugation into plasma and erythrocytes. The plasma was transferred to cryovials, and samples were stored at −80 °C until fatty acid analysis. Lipids were extracted from plasma using a modified procedure based on Folch et al. (1957) [74]. In brief, 4 mL of 2:1 chloroform methanol (*v*/*v*) was added to 400 mL of plasma and sonicated. Four hundred microliters of 0.88% potassium chloride solution was added and the samples were sonicated and centrifuged. The upper organic layer was pipetted off and evaporated to dryness. Plasma phospholipids were isolated by thin layer chromatography using silica gel 60 plates (20 cm × 20 cm; 25 mm thickness). The plates were developed in 60/40/3 hexane/ether/acetic acid (*v*/*v*/*v*) and the phospholipid bands were visualized with dichlorofluorescein. Tricosanoic free fatty acid (Nu-Check Prep, Elysian, MN, USA) was added to each sample as an internal standard. The phospholipids were saponified with 0.5 N methanolic sodium hydroxide and the fatty acids were converted to methyl esters with 14% boron trifluoride methanol at 100 °C for 30 min [75]. All samples were purged with nitrogen throughout the process to minimize oxidation. Fatty acid methyl esters were analyzed by gas–liquid chromatography using a Hewlett Packard 6890 equipped with a flame ionization detector (Agilent Technologies, Santa Clara, CA, USA). The fatty acid methyl esters were separated on a 30 m FAMEWAX capillary column (Restek, Bellefonte, PA, USA; 0.25 mm diameter, 0.25 mm coating thickness) using hydrogen at a flow rate of 2.1 mL/min with a split ratio of 48:1. The chromatographic run parameters included an oven starting temperature of 130 °C that was increased at 6 °C/min to 225 °C, where it was held for 20 min before increasing to 250 °C at 15 °C/min, with a final hold of 5 min. The injector and detector temperatures were constant at 220 °C and 230 °C, respectively. Peaks were identified by comparing retention times with external fatty acid methyl ester standard mixtures from Nu-Check Prep (Elysian, MN, USA). The fatty acid profiles for the plasma phospholipids were expressed as concentration (µg of fatty acid/mL of plasma).

### 4.5. Statistical Analysis Software

Demographics and compliance statistics were performed using the SAS software v9.2 (SAS Institute, Cary, NC, USA). The R statistical software v4.2.3 (Vienna, Austria) was used for all other statistical analyses. All results were considered statistically significant when *p*-values were <0.05. Where indicated, data are presented as mean ± SD.

### 4.6. Statistical Analyses for Demographics and Dietary Intake

Baseline demographics and compliance were analyzed by one-way analysis of variance (ANOVA) for continuous variables (e.g., age and body mass index) or the Cochran–Mantel–Haenszel test for discrete variables (e.g., gender and ethnicity), as previously reported in Maki et al. [40]. DHA and EPA intake from food was determined based on FFQ. The descriptive statistics of the FFQ-derived DHA and EPA intake are presented in Supplementary Table S2. A repeated measures analysis of covariance (ANCOVA) was used to assess the group differences and percent change from baseline in FFQ-derived DHA and EPA intake. Models included the treatment factor, statin use, and baseline value as fixed effects and subjects as a random effect. Pearson’s correlation analysis was conducted to determine the correlation between (1) FFQ-derived DHA and EPA intake and (2) FFQ-derived DHA and EPA intake and the DHA and EPA plasma levels.

### 4.7. Statistical Analyses for Treatment Effect of DHA and EPA Plasma Levels

Repeated measures ANCOVA was used to assess the percent change in total plasma phospholipid DHA+EPA, DHA, and EPA levels from baseline at weeks 6 and 14. Models included the treatment factor, statin use, and baseline value as fixed and subjects as a random effect. The difference between microalgal or fish oil treatment group and placebo group was considered statistically significant when the *p*-value was <0.05, using the difference in the least squares means generated from the final model. The descriptive statistics of the plasma DHA and EPA concentrations before normalization are presented in Supplementary Table S3.

### 4.8. Statistical Analyses for Bioavailability Comparison

To compare the bioavailability of microalgal oil and fish oil, plasma phospholipid DHA and EPA levels were normalized to the daily DHA and EPA intake to account for the potential saturation of plasma DHA and EPA levels, as previously described [55], taking into account the actual number of capsules consumed by each subject. The GMR statistical analysis was used to compare the plasma levels of total DHA+EPA, DHA, and EPA on intake-adjusted data. GMR in microalgal vs. fish oil treatment groups and its 90% CI was estimated as the difference in least squares means from linear mixed effect models on intake-adjusted measures with statin used and treatment as fixed effects and subjects as random effect. The microalgal treatment was considered non-inferior to the fish oil treatment if the lower bound of the 90% CI of the GMR was ≥80%.

### 4.9. Statistical Analyses for a Posteriori Power Calculation

We evaluated power of the findings by a posteriori estimate based on actual sample sizes and observed means and standard deviations. We used a two-sided *t*-test with α = 0.05 for the comparative analyses of microalgal and fish oil treatments vs. placebo. The power analysis for the non-inferiority tests used one-sided *t*-tests on GMR with α = 0.05.

## 5. Conclusions

In conclusion, we have demonstrated that a microalgal-derived omega-3 fatty acid oil can increase total plasma phospholipid DHA and EPA to a level nutritionally non-inferior to fish-derived omega-3 fatty acid oil, despite the different EPA to DHA ratios and doses. Our findings suggest that the established health benefits of omega-3 fatty acids can be leveraged regardless of source, which is noteworthy given that the most evidence to date has been based on fish or fish oil products. Thus, the current study supports the notion that microalgal oil is a sustainable, plan-based and bioavailable source of DHA and EPA that can help us meet the global nutritional needs for these essential macronutrients, similar to fish oil.

## Data Availability

Available upon request from interested researchers.

## Supplementary Materials

The supporting information can be downloaded at https://www.mdpi.com/article/10.3390/ijms26199343/s1.

## Abbreviations

The following abbreviations are used in this manuscript:

DHA	Docosahexaenoic acid
EPA	Eicosapentaenoic acid
LC-PUFA	Long-chain polyunsaturated fatty acids
ALA	Alpha-linolenic acid
RBC	Red blood cell
FFQ	Food frequency questionnaire
ANOVA	Analysis of variance
ANCOVA	Analysis of covariance
GMR	Geometric mean ratio
CI	Confidence interval
