# Supplementary material for: Comparative Bioavailability of DHA and EPA from Microalgal and Fish Oil in Adults
Source: Int J Mol Sci. 2025 Sep 24;26(19):9343. doi: 10.3390/ijms26199343 (PMC12524788; doi:10.3390/ijms26199343)
Supplement: Supplementary file 1 [file ijms-26-09343-s001.zip › ijms-3864894-supplementary.pdf]

Supplementary Materials for *Comparative Bioavailability of DHA and EPA from Microalgal and Fish Oil in Adults*

Eileen Bailey [1,*], Jérôme Wojcik [2], Maike Rahn [1], Franz Roos [1], Anneleen Spooren [1] and Kyoko Koshibu [1,*]

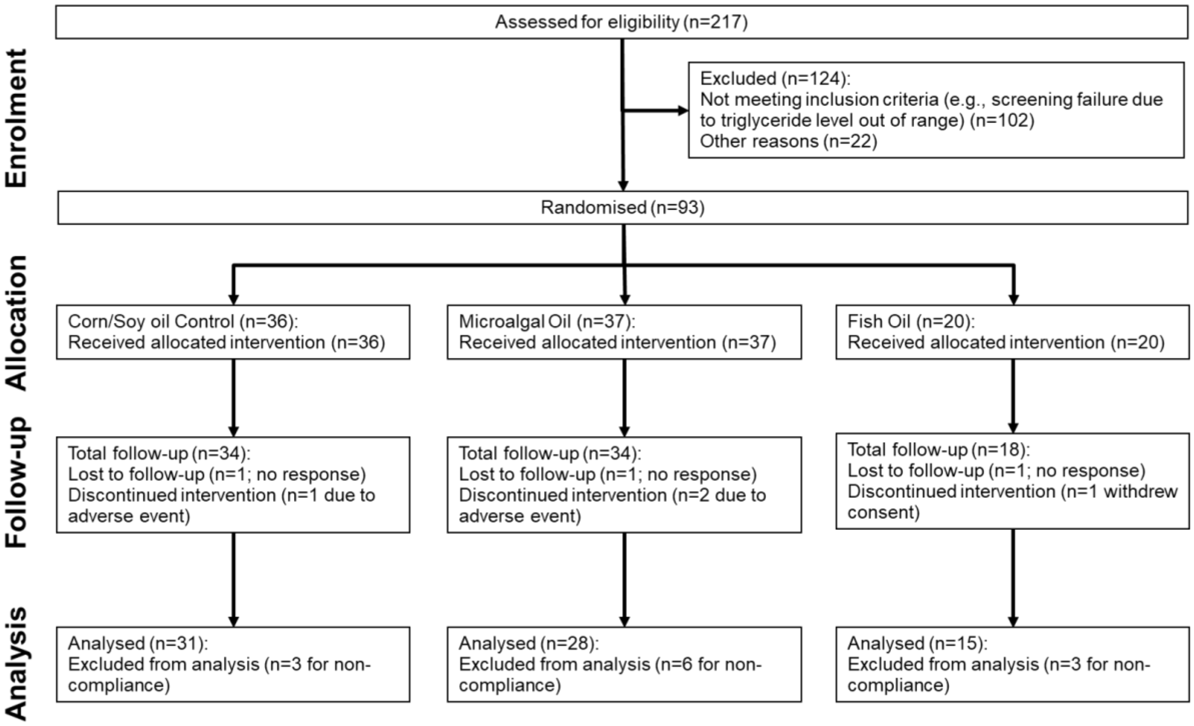

Supplementary Figure S1. CONSORT Diagram for subject disposition in the study

CONSORT Diagram for subject disposition in the study Of 217 subjects recruited, 124 subjects were excluded and 93 subjects were randomized into 3 groups as described in Maki et al. (2014). Data from 74 subjects were analyzed based on non-compliance, loss to follow-up and other criteria.

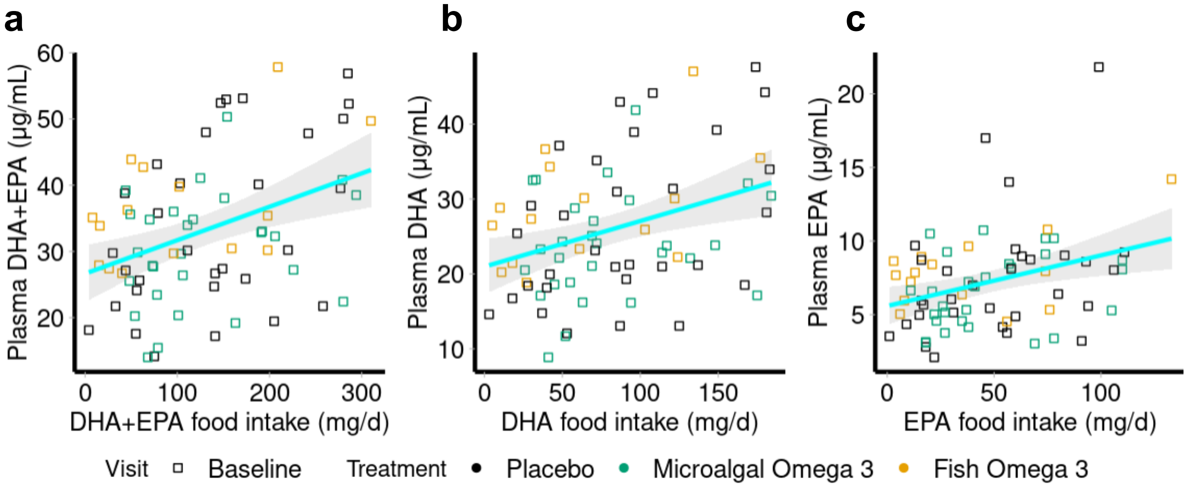

Supplementary Figure S2. Correlation analysis between FFQ estimated DHA and EPA intake and plasma levels of DHA and EPA

The estimated DHA and EPA intake based on FFQ significantly correlated with the plasma levels of (a) DHA + EPA (Pearson's coefficient of correlation: R=0.39, p<0.001), (b) DHA (R=0.35, p<0.01), and (c) EPA (R=0.34, p<0.01). FFQ and plasma data from all visits are combined. Open squares represent FFQ-estimated dose and plasma levels at baseline. Black, green, and orange squares represent FFQ-estimated dose and plasma levels from placebo, microalgal, and fish oils, respectively.

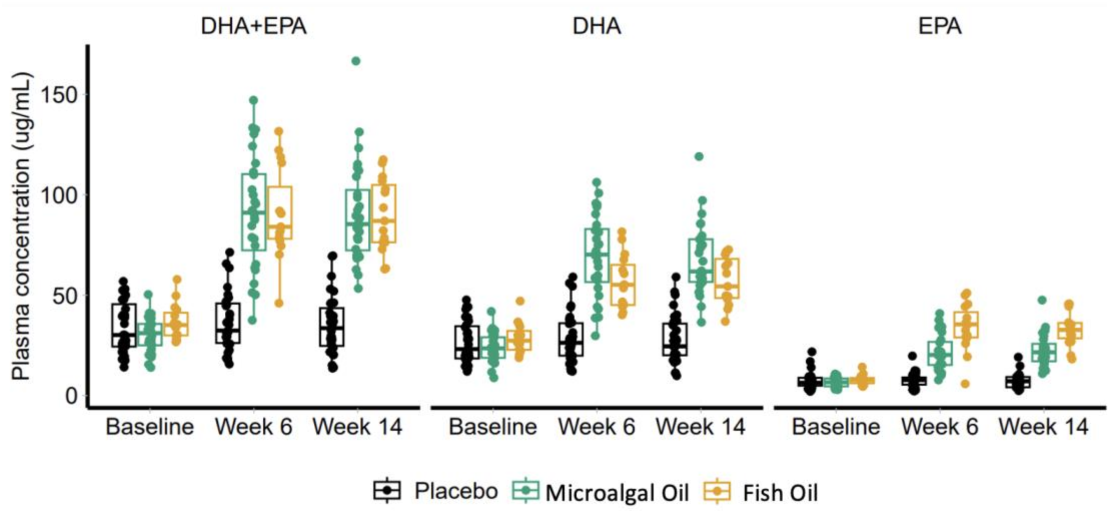

Supplementary Figure S3. Changes in plasma DHA & EPA bioavailability after 6 and 14 weeks

Total plasma DHA and EPA (left), plasma DHA (middle), and plasma EPA (right) levels at baseline, week 6, and week 14 for placebo (black circles and lines), microalgal oil (green circles and lines), and fish oil (orange circle and lines) groups. Each dot represents individual subjects. The DHA and EPA levels are significantly elevated at weeks 6 and 14 for microalgal and fish oil groups, but not for the placebo group. Box plots represent median and 25th and 75th percentile. Data normalized to DHA & EPA intake from diet and supplements, based on the actual pill counts.

Supplementary Table S1. Fatty acid composition of the fish and microalgal oils used in the study

	Placebo	fish	Microalgal
EPA	<1.0 mg/g	280.7 mg/g	164 mg/g
DHA	<1.0 mg/g	199.2 mg/g	443 mg/g
Omega 3 FA	<1.0 mg/g	530.3 mg/g	598 mg/g
Saturated FA			
8:0 Caprylic	<0.10%	<0.10%	<0.10%
10:0 Capric	<0.10%	<0.10%	<0.10%
12:0 Lauric	<0.10%	<0.10%	<0.10%
14:0 Myristic	<0.10%	0.44%	1.5%
15:0 Pentadecanoic	<0.10%	<0.10%	0.2 - 2.0%
16:0 Palmitic	10.88%	2.25%	18.6%
17:0 Margaric	0.14%	0.23%	0.1%
18:0 Stearic	3.06%	3.77%	1.3%
20:0 Arachidic	0.40%	0.49%	0.4%
21:0 Henicosanoic	<0.10%	0.14%	<0.10%
22:0 Behenate	0.26%	0.3%	0.1%
23:0 Tricosanoic	<0.10%	<0.10%	<0.10%
24:0 Lignoceric	0.14%	0.16%	<0.10%
Monosaturated FA			
14:1 Myristoleic	<0.10%	<0.10%	<0.10%
15:1 Pentadecenoic	<0.10%	<0.10%	<0.10%
16:1 Palmitoleic	0.14%	1.02%	<0.10%
17:1 Heptadecenoic	<0.10%	0.12%	<0.10%
18:1n-9 Oleic Acid	28.14%	12.07%	3.5%
18:1n-7 Vaccenic	1.14%	2.34%	0.1%
20:1n-9 Eicosenoic	0.55%	2.58%	<0.10%
22:1n-9 Erucic	<0.10%	2.62%	<0.10%
24:1 Nervonic	<0.10%	0.61%	<0.10%
Polyunsaturated FA			
18:2n-6 Linoleic	49.53%	1.55%	0.6%
18:3n-3 α-Linolenic	2.67%	0.72%	<0.1%
18:3n-6 Ƴ-Linolenic	0.61%	0.17%	<0.10%
20:2n-6 Eicosadienoic	<0.10%	1.89%	<0.10%
20:3n-3	<0.10%	0.16%	<0.10%
20:3n-6 dihomo-Ƴ-Linolenic	<0.10%	0.43%	<0.10%
20:4n-6 Arachidonic	<0.10%	1.75%	1.3%
20:5n-3 EPA	<0.10%	31.10%	19.8%
22:2n-6 Docosadienoate	<0.10%	1.48%	<0.10%
22:5n-3 DPAn-3	<0.10%	3.89%	4.7%
22:5n-6 DPAn-6	<0.10%	0.84%	1.3%
C22:6n-3 DHA	<0.10%	22.31%	35.2%

Supplementary Table S2. DHA and EPA intake from food based on FFQ

		Placebo	Microalgal Omega 3	Fish Omega 3
DHA + EPA intake (mg/d)				

Baseline	N<br>Mean (SD)<br>Median [range]	36<br>142.0 (84.2)<br>141.0 [4.0 - 286.0]	37<br>122.8 (75.7)<br>101.0 [27.0 - 294.0]	20<br>114.8 (95.9)<br>85.5 [8.0 - 310.0]
Week 6	N<br>Mean (SD)<br>Median [range]	34<br>161.6 (103.6)<br>144.5 [33.0 - 513.0]	34<br>117.4 (68.7)<br>98.5 [14.0 - 294.0]	20<br>116.1 (97.2)<br>82.0 [7.0 - 311.0]
Week 14	N<br>Mean (SD)<br>Median [range]	34<br>143.1 (77.2)<br>143.0 [3.0 - 345.0]	34<br>139.1 (78.6)<br>127.5 [22.0 - 319.0]	20<br>121.9 (95.6)<br>104.0 [9.0 - 297.0]
DHA intake (mg/d)				
Baseline	N<br>Mean (SD)<br>Median [range]	36<br>90.2 (52.9)<br>87.0 [3.0 - 183.0]	37<br>77.6 (47.1)<br>68.0 [18.0 - 184.0]	20<br>72.4 (58.1)<br>54.5 [5.0 - 177.0]
Week 6	N<br>Mean (SD)<br>Median [range]	34<br>102.7 (65.6)<br>93.5 [18.0 - 324.0]	34<br>73.9 (42.1)<br>79.0 [8.0 - 184.0]	20<br>74.6 (61.1)<br>55.5 [12.0 - 189.0]
Week 14	N<br>Mean (SD)<br>Median [range]	34<br>92.5 (50.6)<br>92.0 [3.0 - 224.0]	34<br>88.4 (49.3)<br>79.0 [10.0 - 200.0]	20<br>77.9 (60.3)<br>49.0 [1.0 - 121.0]
EPA intake (mg/d)				
Baseline	N<br>Mean (SD)<br>Median [range]	36<br>51.8 (31.7)<br>55.0 [1.0 - 111.0]	37<br>45.2 (29.0)<br>37.0 [9.0 - 110.0]	20<br>42.4 (38.2)<br>31.0 [3.0 - 133.0]
Week 6	N<br>Mean (SD)<br>Median [range]	34<br>58.9 (38.7)<br>54.5 [12.0 - 189.0]	34<br>43.6 (27.3)<br>38.0 [6.0 - 110.0]	20<br>41.2 (36.3)<br>30.0 [1.0 - 113.0]
Week 14	N<br>Mean (SD)<br>Median [range]	34<br>50.6 (30.1)<br>49.0 [1.0 - 121.0]	34<br>50.7 (30.0)<br>47.0 [10.0 - 119.0]	20<br>44.0 (55.6)<br>37.5 [2.0 - 105.0]
EPA:DHA ratio				
Baseline	N<br>Mean (SD)<br>Median [range]	36<br>0.56 (0.10)<br>0.57 [0.33 - 0.83]	37<br>0.58 (0.11)<br>0.58 [0.33 - 0.90]	20<br>0.54 (0.11)<br>0.56 [0.28 - 0.75]
Week 6	N<br>Mean (SD)<br>Median [range]	34<br>0.58 (0.09)<br>0.58 [0.37 - 0.83]	34<br>0.58 (0.13)<br>0.59 [0.29 - 0.84]	20<br>0.52 (0.13)<br>0.57 [0.17 - 0.61]
Week 14	N<br>Mean (SD)<br>Median [range]	34<br>0.55 (0.14)<br>0.59 [0.06 - 0.83]	34<br>0.58 (0.15)<br>0.60 [0.33 - 1.20]	20<br>0.54 (0.09)<br>0.56 [0.29 - 0.64]

Supplementary Table S3. Plasma DHA and EPA concentrations before normalization to intake of dietary supplements

		Placebo	Microalgal Oil	Fish Oil
Plasma DHA + EPA (µg/mL)				
Baseline	N<br>Mean (SD)<br>Median [range]	31<br>34.0 (12.9)<br>30.2 [14.2 - 56.9]	28<br>30.4 (8.5)<br>31.1 [14.0 - 50.3]	15<br>36.5 (8.9)<br>35.1 [26.7 - 57.8]
Week 6	N<br>Mean (SD)<br>Median [range]	31<br>36.6 (14.4)<br>32.4 [15.7 - 71.4]	28<br>91.1 (28.0)<br>91.1 [37.5 - 147.1]	15<br>90.4 (22.8)<br>84.0 [46.0 - 131.6]

Week 14	N<br>Mean (SD)<br>Median [range]	31<br>35.3 (14.7)<br>33.6 [13.6 - 69.6]	28<br>90.4 (24.7)<br>85.4 [53.4 - 166.6]	15<br>89.7 (18.3)<br>87.0 [63.0 - 117.5]
Plasma DHA (μg/mL)				
Baseline	N<br>Mean (SD)<br>Median [range]	31<br>26.5 (10.5)<br>23.1 [12.1 - 47.6]	28<br>23.8 (9.9)<br>23.5 [8.9 - 41.9]	15<br>28.6 (7.5)<br>27.3 [18.9 - 47.1]
Week 6	N<br>Mean (SD)<br>Median [range]	31<br>28.9 (12.6)<br>26.3 [12.1 - 59.1]	28<br>69.5 (20.4)<br>70.2 [29.8 - 106.2]	15<br>56.5 (13.3)<br>54.4 [36.9 - 72.7]
Week 14	N<br>Mean (SD)<br>Median [range]	31<br>28.0 (12.2)<br>24.5 [9.9 - 59.0]	28<br>67.7 (17.6)<br>61.8 [36.5 - 119.0]	15<br>56.8 (11.9)<br>54.4 [36.9 - 72.7]
Plasma EPA (μg/mL)				
Baseline	N<br>Mean (SD)<br>Median [range]	31<br>7.4 (4.1)<br>6.4 [2.1 - 21.8]	28<br>6.6 (2.5)<br>6.6 [3.0 - 10.7]	15<br>7.9 (2.5)<br>7.9 [4.5 - 14.2]
Week 6	N<br>Mean (SD)<br>Median [range]	31<br>7.8 (3.5)<br>7.7 [2.3 - 19.7]	28<br>21.6 (8.9)<br>20.3 [7.8 - 40.9]	15<br>33.9 (11.8)<br>35.4 [5.8 - 51.1]
Week 14	N<br>Mean (SD)<br>Median [range]	31<br>7.2 (3.6)<br>7.2 [2.4 - 19.1]	28<br>22.7 (8.0)<br>21.5 [10.9 - 47.6]	15<br>32.9 (8.3)<br>32.7 [18.2 - 45.7]

Supplementary Table S4. Treatment effect of microalgal and fish oil on plasma DHA and EPA

**Linear mixed effect model [Estimate (SE)]**					
**Biomarker**	**intercept**	**Baseline**	**Statin**	**Treatment Microalgal**	**Treatment Fish**
DHA+EPA	62.6 (22.1)<br>p = 0.0050	-1.6 (0.6)<br>p = 0.0058	-17.6 (24.1)<br>p = 0.4667	129.0 (14.0)<br>p < 0.0001	106.9 (17.3)<br>p < 0.0001
DHA	59.6 (19.6)<br>p = 0.0026	-2.0 (0.7)<br>p = 0.0028	-9.8 (22.4)<br>p = 0.6621	124.4 (13.0)<br>p < 0.0001	71.9 (16.1)<br>p < 0.0001
EPA	101.2 (32.7)<br>p = 0.0029	-12.2 (3.7)<br>p = 0.0014	-49.6 (46.5)<br>p = 0.2902	165.7 (26.7)<br>p < 0.0001	247.5 (33.2)<br>p < 0.0001
**ANCOVA**					
**Biomarker**	**df**	**N**	**Chi2**	***p*-value**	
DHA+EPA	2	74	96.01	p < 0.0001	
DHA	2	74	94.04	p < 0.0001	
EPA	2	74	70.75	p < 0.0001	

Supplementary Table S5. Percent change from baseline in plasma DHA and EPA concentrations before normalization to intake of dietary supplements

		**Placebo**	**Microalgal Oil**	**Fish Oil**
Plasma DHA + EPA (μg/mL)				

Week 6	N<br>Mean (SD)<br>Median [range]	31<br>10% (24)<br>4% [-42 - 54%]	28<br>212% (106)<br>182% [92 - 563%]	15<br>161% (89)<br>163% [29 - 332%]
Week 14	N<br>Mean (SD)<br>Median [range]	31<br>7% (31)<br>6% [-47 - 105%]	28<br>213% (102)<br>199% [77 - 531%]	15<br>154% (61)<br>236% [79 - 280%]
Plasma DHA (µg/mL)				
Week 6	N<br>Mean (SD)<br>Median [range]	31<br>10% (26)<br>7% [-33 - 69%]	28<br>207% (107)<br>189% [79 - 604%]	15<br>109% (69)<br>92% [17 - 250%]
Week 14	N<br>Mean (SD)<br>Median [range]	31<br>8% (29)<br>10% [-44 - 92%]	28<br>203% (98)<br>179% [53 - 508%]	15<br>107% (59)<br>82% [42 - 259%]
Plasma EPA (µg/mL)				
Week 6	N<br>Mean (SD)<br>Median [range]	31<br>15% (38)<br>16% [-68 - 89%]	28<br>270% (229)<br>198% [51 - 993%]	15<br>369% (254)<br>315% [16 - 1003%]
Week 14	N<br>Mean (SD)<br>Median [range]	31<br>6% (46)<br>-2% [-58 - 147%]	28<br>289% (206)<br>215% [55 - 856%]	15<br>348% (185)<br>293% [153 - 905%]

Supplementary Table S6. Changes in plasma DHA and EPA normalized to intake of dietary supplements

		Microalgal Oil	Fish Oil
Plasma DHA + EPA (µg/mL/mg intake/day)			
Week 6	N<br>Mean (SD)<br>Median [range]	28<br>2.11 (0.70)<br>2.13 [0.77 - 3.63]	15<br>1.85 (0.50)<br>1.78 [0.78 - 2.69]
Week 14	N<br>Mean (SD)<br>Median [range]	28<br>2.13 (0.63)<br>2.02 [1.25 - 4.10]	15<br>1.82 (0.38)<br>1.86 [1.18 - 2.46]
Plasma DHA (µg/mL /mg intake/day)			
Week 6	N<br>Mean (SD)<br>Median [range]	28<br>1.03 (0.30)<br>1.04 [0.43 - 1.57]	15<br>0.88 (0.21)<br>0.86 [0.62 - 1.27]
Week 14	N<br>Mean (SD)<br>Median [range]	28<br>1.00 (0.26)<br>0.91 [0.53 - 1.76]	15<br>0.89 (0.19)<br>0.85 [0.58 - 1.13]
Plasma EPA (µg/mL /mg intake/day)			
Week 6	N<br>Mean (SD)<br>Median [range]	28<br>1.08 (0.44)<br>1.02 [0.33 - 2.06]	15<br>0.96 (0.35)<br>1.00 [0.15 - 1.49]
Week 14	N<br>Mean (SD)<br>Median [range]	28<br>1.13 (0.40)1.13<br>[0.54 - 2.35]	15<br>0.93 (0.24)0.96 [0.48 -<br>1.33]

Supplementary Table S7. Total plasma phospholipid analysis as a percentage of total fatty acid

Fatty Acid	Visit	Placebo N, mean (SD) median[min-max]	Microalgal Omega 3 N, mean (SD) median[min-max]	Fish Omega 3 N, mean (SD) median[min-max]
14:0	Baseline	N=31, 0.25 (0.07) 0.26 [0.12-0.38]	N=28, 0.26 (0.07) 0.26 [0.14-0.4]	N=15, 0.27 (0.1) 0.27 [0.12-0.51]
	Week 6	N=31, 0.28 (0.08) 0.28 [0.12-0.44]	N=28, 0.26 (0.08) 0.27 [0.09-0.45]	N=15, 0.28 (0.08) 0.3 [0.15-0.42]
	Week 14	N=31, 0.34 (0.13) 0.3 [0.18-0.72]	N=28, 0.33 (0.1) 0.31 [0.14-0.54]	N=15, 0.28 (0.13) 0.24 [0.16-0.65]
15:0	Baseline	N=31, 0.16 (0.03) 0.17 [0.09-0.22]	N=28, 0.17 (0.03) 0.17 [0.09-0.23]	N=15, 0.16 (0.05) 0.18 [0.09-0.25]
	Week 6	N=31, 0.17 (0.04) 0.16 [0.08-0.24]	N=28, 0.17 (0.03) 0.17 [0.09-0.24]	N=15, 0.16 (0.04) 0.17 [0.09-0.23]
	Week 14	N=31, 0.18 (0.04) 0.18 [0.11-0.28]	N=28, 0.18 (0.03) 0.18 [0.11-0.27]	N=15, 0.17 (0.06) 0.16 [0.09-0.33]
16:0	Baseline	N=31, 26.83 (1.3) 26.94 [24.22-29.44]	N=28, 26.59 (1.68) 26.71 [23.42-30.1]	N=15, 26.99 (1.95) 25.95 [25.24-31.52]
	Week 6	N=31, 26.52 (1.5) 26.24 [24.63-30.61]	N=28, 27.03 (1.62) 26.8 [24.35-30.68]	N=15, 27 (1.68) 26.61 [24.94-30.21]
	Week 14	N=31, 26.72 (1.52) 26.53 [24.37-29.78]	N=28, 27.39 (1.4) 27.06 [24.97-30.73]	N=15, 27.19 (1.7) 27.32 [24.45-30.83]
16:1	Baseline	N=31, 0.51 (0.14) 0.47 [0.23-0.81]	N=28, 0.57 (0.26) 0.54 [0.26-1.52]	N=15, 0.59 (0.28) 0.49 [0.2-1.05]
	Week 6	N=31, 0.55 (0.16) 0.54 [0.22-0.97]	N=28, 0.49 (0.21) 0.46 [0.22-1.24]	N=15, 0.47 (0.26) 0.34 [0.15-1.12]
	Week 14	N=31, 0.55 (0.2) 0.53 [0.3-1.26]	N=28, 0.54 (0.26) 0.5 [0.27-1.42]	N=15, 0.51 (0.27) 0.42 [0.15-1.23]
17:0	Baseline	N=31, 0.36 (0.06) 0.37 [0.2-0.48]	N=28, 0.36 (0.05) 0.36 [0.19-0.43]	N=15, 0.35 (0.08) 0.37 [0.2-0.44]
	Week 6	N=31, 0.36 (0.07) 0.36 [0.2-0.51]	N=28, 0.36 (0.07) 0.36 [0.19-0.52]	N=15, 0.36 (0.09) 0.39 [0.16-0.48]
	Week 14	N=31, 0.37 (0.07) 0.36 [0.22-0.54]	N=28, 0.37 (0.07) 0.37 [0.21-0.55]	N=15, 0.37 (0.08) 0.37 [0.21-0.48]
18:0	Baseline	N=31, 15.9 (1.18) 15.81 [13.43-18.12]	N=28, 15.64 (1.68) 15.97 [11.25-18.32]	N=15, 15.44 (1.36) 15.79 [12.51-17.15]
	Week 6	N=31, 15.62 (1.24) 15.69 [12.83-17.56]	N=28, 15.85 (1.5) 15.94 [11.48-19.16]	N=15, 15.71 (1.2) 16.04 [13.31-17.39]
	Week 14	N=31, 15.87 (1.1) 15.87 [13.26-18.3]	N=28, 15.82 (1.19) 15.86 [12.92-18]	N=15, 15.91 (1.43) 16.18 [13.29-18.08]
18:1 n-7	Baseline	N=31, 1.57 (0.24) 1.58 [1.2-2.11]	N=28, 1.8 (0.37) 1.65 [1.27-2.79]	N=15, 1.66 (0.24) 1.68 [1.27-1.96]
	Week 6	N=31, 1.56 (0.26) 1.49 [1.01-2.25]	N=28, 1.67 (0.42) 1.6 [1.04-2.79]	N=15, 1.5 (0.12) 1.49 [1.3-1.68]
	Week 14	N=31, 1.57 (0.28) 1.53 [1.12-2.3]	N=28, 1.68 (0.43) 1.56 [1.17-3.07]	N=15, 1.53 (0.2) 1.54 [1.27-1.93]
18:1 n-9	Baseline	N=31, 8.43 (1.07) 8.24 [6.43-10.3]	N=28, 8.7 (1.19) 8.56 [6.94-12.33]	N=15, 8.86 (0.51) 8.84 [7.79-9.55]

	Week 6	N=31, 8.47 (1.03)<br>8.28 [6.68-11.39]	N=28, 7.98 (1.06)<br>7.9 [6.46-10.06]	N=15, 7.83 (0.75)<br>7.6 [6.97-9.66]
	Week 14	N=31, 8.58 (1.04)<br>8.61 [6.64-11.25]	N=28, 8.18 (1.09)<br>8.11 [6.49-11.06]	N=15, 8.11 (0.95)<br>8.12 [6.72-9.57]
18:2	Baseline	N=31, 23.05 (3.27)<br>22.67 [17.19-30.64]	N=28, 23.07 (2.47)<br>22.67 [18.03-28.43]	N=15, 23.09 (3.56)<br>22.73 [17.09-28.7]
	Week 6	N=31, 23.1 (2.98)<br>23.06 [16.52-28.89]	N=28, 20.3 (2.35)<br>20.23 [15.23-25.08]	N=15, 20.95 (3.11)<br>21.06 [15.68-26.11]
	Week 14	N=31, 23.28 (3.25)<br>23.06 [16.26-29.84]	N=28, 20.32 (2.28)<br>20.47 [15.25-25.36]	N=15, 20.24(3.16)<br>20.71 [14.92-25.98]
18:3 n-3	Baseline	N=31, 0.22 (0.06)<br>0.21 [0.12-0.37]	N=28, 0.22 (0.06)<br>0.22 [0.13-0.4]	N=15, 0.21 (0.05)<br>0.2 [0.09-0.29]
	Week 6	N=31, 0.21 (0.06)<br>0.22 [0.12-0.34]	N=28, 0.2 (0.06)<br>0.19 [0.11-0.36]	N=15, 0.2 (0.06)<br>0.19 [0.11-0.31]
	Week 14	N=31, 0.26 (0.07)<br>0.25 [0.11-0.46]	N=28, 0.21 (0.05)<br>0.21 [0.11-0.33]	N=15, 0.21 (0.06)<br>0.22 [0.1-0.32]
18:3 n-6	Baseline	N=31, 0.12 (0.04)<br>0.11 [0.06-0.23]	N=28, 0.12 (0.06)<br>0.13 [0-0.31]	N=15, 0.12 (0.05)<br>0.1 [0.04-0.2]
	Week 6	N=31, 0.14 (0.06)<br>0.13 [0.05-0.28]	N=28, 0.09 (0.05)<br>0.08 [0-0.17]	N=15, 0.07 (0.03)<br>0.06 [0.03-0.12]
	Week 14	N=31, 0.14 (0.07)<br>0.12 [0.04-0.29]	N=28, 0.09 (0.06)<br>0.08 [0-0.31]	N=15, 0.08 (0.04)<br>0.06 [0.04-0.19]
20:0	Baseline	N=31, 0.13 (0.05)<br>0.12 [0.05-0.24]	N=28, 0.14 (0.06)<br>0.13 [0.07-0.29]	N=15, 0.12 (0.06)<br>0.1 [0-0.25]
	Week 6	N=31, 0.13 (0.04)<br>0.13 [0.07-0.22]	N=28, 0.15 (0.05)<br>0.15 [0.08-0.3]	N=15, 0.13 (0.04)<br>0.13 [0.08-0.25]
	Week 14	N=31, 0.12 (0.04)<br>0.12 [0.06-0.21]	N=28, 0.11 (0.04)<br>0.11 [0.05-0.2]	N=15, 0.11 (0.04)<br>0.1 [0.07-0.2]
20:1 n-9	Baseline	N=31, 0.12 (0.03)<br>0.11 [0.08-0.18]	N=28, 0.12 (0.03)<br>0.11 [0.08-0.19]	N=15, 0.12 (0.03)<br>0.11 [0.08-0.19]
	Week 6	N=31, 0.12 (0.03)<br>0.12 [0.08-0.22]	N=28, 0.11 (0.04)<br>0.1 [0.08-0.27]	N=15, 0.12 (0.03)<br>0.12 [0.09-0.18]
	Week 14	N=31, 0.12 (0.03)<br>0.11 [0.08-0.2]	N=28, 0.1 (0.02)<br>0.1 [0.07-0.15]	N=15, 0.12 (0.02)<br>0.11 [0.08-0.16]
20:2	Baseline	N=31, 0.35 (0.06)<br>0.36 [0.23-0.44]	N=28, 0.36 (0.07)<br>0.34 [0.27-0.52]	N=15, 0.34 (0.05)<br>0.33 [0.28-0.45]
	Week 6	N=31, 0.37 (0.07)<br>0.37 [0.23-0.51]	N=28, 0.35 (0.07)<br>0.34 [0.18-0.51]	N=15, 0.32 (0.06)<br>0.32 [0.26-0.51]
	Week 14	N=31, 0.36 (0.04)<br>0.36 [0.27-0.46]	N=28, 0.34 (0.05)<br>0.34 [0.27-0.48]	N=15, 0.32 (0.04)<br>0.33 [0.22-0.37]
20:3 n-3	Baseline	N=31, 0(0) 0[0-0]	N=28, 0(0) 0[0-0]	N=15, 0(0) 0[0-0]
	Week 6	N=31, 0(0) 0[0-0]	N=28, 0(0) 0[0-0]	N=15, 0(0) 0[0-0]
	Week 14	N=31, 0(0) 0[0-0]	N=28, 0(0) 0[0-0]	N=15, 0(0) 0[0-0]
20:3 n-6	Baseline	N=31, 3.86 (0.78)<br>3.8 [2.42-5.61]	N=28, 4.02 (0.85)<br>3.9 [2.33-5.82]	N=15, 3.72 (0.93)<br>3.51 [2.07-5.4]
	Week 6	N=31, 4.05 (0.95)<br>3.99 [2.35-6.65]	N=28, 3.26 (0.75)<br>3.4 [1.27-4.73]	N=15, 2.93 (0.72)<br>2.8 [1.66-4.32]

	Week 14	N=31, 3.86 (0.83)<br>3.72 [2.66-5.95]	N=28, 3.21 (0.76)<br>3.15 [1.65-5.6]	N=15, 2.9 (0.73)<br>2.88 [1.89-4.42]
20:3 n-9	Baseline	N=31, 0.1 (0.05)<br>0.1 [0-0.25]	N=28, 0.13 (0.08)<br>0.13 [0-0.44]	N=15, 0.11 (0.04)<br>0.12 [0-0.16]
	Week 6	N=31, 0.11 (0.05)<br>0.1 [0-0.24]	N=28, 0.08 (0.04)<br>0.07 [0-0.17]	N=15, 0.06 (0.03)<br>0.06 [0-0.12]
	Week 14	N=31, 0.1 (0.05)<br>0.09 [0-0.27]	N=28, 0.08 (0.05)<br>0.07 [0-0.2]	N=15, 0.06 (0.04)<br>0.06 [0-0.15]
22:0	Baseline	N=31, 0.2 (0.11)<br>0.2 [0-0.51]	N=28, 0.19 (0.09)<br>0.18 [0-0.38]	N=15, 0.18 (0.1)<br>0.16 [0-0.36]
	Week 6	N=31, 0.23 (0.1)<br>0.23 [0-0.5]	N=28, 0.29 (0.11)<br>0.27 [0.13-0.59]	N=15, 0.23 (0.1)<br>0.23 [0.1-0.45]
	Week 14	N=31, 0.17 (0.12)<br>0.18 [0-0.52]	N=28, 0.16 (0.09)<br>0.18 [0-0.35]	N=15, 0.15 (0.11)<br>0.15 [0-0.35]
22:2	Baseline	N=31 0(0) 0[0-0]	N=28, 0(0) 0[0-0]	N=15, 0(0) 0[0-0]
	Week 6	N=31 0(0) 0[0-0]	N=28, 0(0) 0[0-0]	N=15, 0(0) 0[0-0]
	Week 14	N=31 0(0) 0[0-0]	N=28, 0(0) 0[0-0]	N=15, 0(0) 0[0-0]
22:4 n-6	Baseline	N=31, 0.45 (0.11)<br>0.45 [0.25-0.71]	N=28, 0.51 (0.12)<br>0.51 [0.25-0.75]	N=15, 0.4 (0.11)<br>0.39 [0.23-0.69]
	Week 6	N=31, 0.48 (0.12)<br>0.48 [0.25-0.79]	N=28, 0.27 (0.07)<br>0.26 [0.16-0.47]	N=15, 0.22 (0.09)<br>0.21 [0-0.35]
	Week 14	N=31, 0.45 (0.1)<br>0.42 [0.23-0.73]	N=28, 0.24 (0.09)<br>0.23 [0-0.51]	N=15, 0.18 (0.11)<br>0.17 [0-0.33]
22:5 n-3	Baseline	N=31, 0.96 (0.18)<br>0.93 [0.64-1.28]	N=28, 0.97 (0.21)<br>0.94 [0.56-1.54]	N=15, 0.88 (0.21)<br>0.92 [0.41-1.27]
	Week 6	N=31, 0.97 (0.18)<br>0.98 [0.6-1.46]	N=28, 1.03 (0.2)<br>1.02 [0.72-1.62]	N=15, 1.29 (0.18)<br>1.28 [0.98-1.67]
	Week 14	N=31, 0.95 (0.21)<br>0.91 [0.57-1.48]	N=28, 0.99 (0.16)<br>1.01 [0.66-1.24]	N=15, 1.32 (0.16)<br>1.29 [1.09-1.67]
22:5 n-6	Baseline	N=31, 0.31 (0.09)<br>0.31 [0-0.48]	N=28, 0.34 (0.1)<br>0.34 [0.19-0.65]	N=15, 0.28 (0.17)<br>0.27 [0-0.62]
	Week 6	N=31, 0.34 (0.12)<br>0.33 [0-0.69]	N=28, 0.21 (0.05)<br>0.21 [0-0.27]	N=15, 0.15 (0.11)<br>0.18 [0-0.33]
	Week 14	N=31, 0.32 (0.11)<br>0.3 [0-0.6]	N=28, 0.21 (0.09)<br>0.2 [0-0.46]	N=15, 0.11 (0.1)<br>0.14 [0-0.29]
24:0	Baseline	N=31, 0.1 (0.12)<br>0 [0-0.41]	N=28, 0.06 (0.09)<br>0 [0-0.26]	N=15, 0.08 (0.11)<br>0 [0-0.3]
	Week 6	N=31, 0.13 (0.13)<br>0.18 [0-0.4]	N=28, 0.2 (0.13)<br>0.23 [0-0.43]	N=15, 0.11 (0.14)<br>0 [0-0.41]
	Week 14	N=31, 0.1 (0.12)<br>0 [0-0.41]	N=28, 0.08 (0.1)<br>0 [0-0.33]	N=15, 0.09 (0.11)<br>0 [0-0.32]
24:1	Baseline	N=31, 0.15 (0.17)<br>0.15 [0-0.66]	N=28, 0.12 (0.15)<br>0 [0-0.46]	N=15, 0.19 (0.19)<br>0.18 [0-0.54]
	Week 6	N=31, 0.22 (0.13)<br>0.22 [0-0.45]	N=28, 0.28 (0.16)<br>0.27 [0-0.7]	N=15, 0.18 (0.23)<br>0 [0-0.65]
	Week 14	N=31, 0.12 (0.14)<br>0 [0-0.44]	N=28, 0.14 (0.12)<br>0.17 [0-0.33]	N=15, 0.14 (0.15)<br>0.15 [0-0.4]

ARA	Baseline	N=31, 12.18 (2.21)<br>12.19 [8.05-18.68]	N=28, 12.27 (1.82)<br>12.06 [9.26-15.99]	N=15, 12.25 (2.36)<br>13.04 [8.68-16.47]
	Week 6	N=31, 12.19 (2.23)<br>12.29 [7.88-16.22]	N=28, 9.98 (1.5)<br>9.61 [6.52-13.21]	N=15, 10.25 (1.79)<br>10.4 [7.15-14.06]
	Week 14	N=31, 11.8 (1.95)<br>11.75 [8.39-15.71]	N=28, 9.54 (1.47)<br>9.52 [6.94-13.35]	N=15, 10.07 (2.38)<br>9.96 [6.77-16.13]
DHA	Baseline	N=31, 2.91 (1)<br>2.65 [1.49-5.21]	N=28, 2.56 (0.75)<br>2.46 [1.13-4.15]	N=15, 2.85 (0.97)<br>2.79 [1.57-5.5]
	Week 6	N=31, 2.9 (1.05)<br>2.73 [1.39-5.29]	N=28, 7.18 (1.65)<br>7.09 [3.65-10.5]	N=15, 5.93 (1.19)<br>5.61 [4.12-8.38]
	Week 14	N=31, 2.94 (1.02)<br>2.68 [1.37-5.25]	N=28, 7.29 (1.42)<br>7.24 [3.83-9.98]	N=15, 6.22 (0.93)<br>6.13 [5.14-7.96]
EPA + DHA	Baseline	N=31, 3.71 (1.17)<br>3.33 [2.12-6.22]	N=28, 3.26 (0.84)<br>3.07 [1.79-4.98]	N=15, 3.62 (1.14)<br>3.54 [2.16-6.77]
	Week 6	N=31, 3.67 (1.17)<br>3.63 [1.88-6.18]	N=28, 9.4 (2.24)<br>9.32 [4.6-13.8]	N=15, 9.49 (2.21)<br>8.91 [6.29-13.79]
	Week 14	N=31, 3.69 (1.22)<br>3.46 [1.93-7.26]	N=28, 9.7 (1.87)<br>9.1 [5.6-13.97]	N=15, 9.82 (1.3)<br>9.53 [8.1-12.41]
EPA	Baseline	N=31, 0.8 (0.38)<br>0.73 [0.32-2.26]	N=28, 0.7 (0.23)<br>0.68 [0.36-1.19]	N=15, 0.77 (0.25)<br>0.63 [0.49-1.26]
	Week 6	N=31, 0.78 (0.3)<br>0.75 [0.29-1.71]	N=28, 2.22 (0.78)<br>2.11 [0.95-3.9]	N=15, 3.56 (1.31)<br>3.45 [0.8-5.42]
	Week 14	N=31, 0.75 (0.33)<br>0.73 [0.33-2]	N=28, 2.41 (0.63)<br>2.3 [1.5-3.99]	N=15, 3.6 (0.74)<br>3.54 [2.63-5.05]
